# Tanshinone IIA inhibits malignant progression in a spontaneous breast cancer mouse model by attenuating tissue factor-mediated angiogenesis

**DOI:** 10.3389/fphar.2026.1848311

**Published:** 2026-07-09

**Authors:** Yuan Wang, Xiaoyue Yan, Zheng Wang, Ye Sun, Ziyan Zhu, Ting Mao, Quanyue Wang, Xiaofeng Fan, Xinyu Lu, Junde Xu, Xiaoman Li, Yin Lu, Yuanyuan Wu

**Affiliations:** 1 Jiangsu Key Laboratory for Pharmacology and Safety Research of Chinese Materia Medica, School of Pharmacy, Nanjing University of Chinese Medicine, Nanjing, Jiangsu, China; 2 State Key Laboratory of Mechanism and Quality of Chinese Medicine, Macau University of Science and Technology, Taipa, China

**Keywords:** angiogenic switch, breast cancer prevention, malignant transformation, tanshinone IIA, tissue factor

## Abstract

**Introduction:**

As a classic botanical drug, *Salvia miltiorrhiza* Bunge has long been used to promote blood circulation and remove blood stasis. Tanshinone IIA (Tan IIA), a major bioactive diterpenoid derived from its roots, shows promising therapeutic potential, but its chemopreventive actions against early tumor progression have not been fully clarified. This study aims to comprehensively examine the chemopreventive potential of Tan IIA, specifically focusing on the poorly studied adenoma-to-early-carcinoma transition in breast cancer, and elucidate its underlying anti-angiogenic mechanisms.

**Methods:**

We adopted the mouse mammary tumor virus-polyoma virus middle T antigen (MMTV-PyMT) transgenic mouse model, which recapitulates the natural progression of spontaneous breast tumorigenesis. A combination of pharmacokinetic analysis, network pharmacology, *in vivo* histopathology, immunofluorescence (IF) staining, multiple angiogenesis assays, and molecular experiments, including qPCR, Western blot, and cellular thermal shift assay (CETSA), was applied for comprehensive evaluation.

**Results:**

Our results demonstrated that Tan IIA markedly delayed the malignant transformation of benign mammary tumors. Pharmacokinetic characterization confirmed the favorable *in vivo* bioavailability of Tan IIA and its metabolites. Mechanistically, Tan IIA significantly downregulated tissue factor (TF), a critical driver of tumor angiogenesis. This reduction in TF expression was accompanied by decreased levels of vascular endothelial growth factor A (VEGFA) and other pro-angiogenic factors.

**Discussion:**

Our work reveals that Tan IIA exerts prominent chemopreventive effects against breast precancerous lesions, mainly by suppressing TF-mediated angiogenesis, indicating its great promise as a candidate agent for early breast cancer intervention.

## Introduction

Breast cancer has emerged as the leading cause of morbidity and mortality among women worldwide (Siegel et al., 2024). More than 25% of women between the ages of 40 and 50 present with benign breast nodules *in situ*, yet less than 1% in this age group are diagnosed with breast cancer (Howlader et al., 2013). This discrepancy indicates that not all *in situ* breast tumors inevitably progress to malignancy. Understanding these mechanisms underlying the malignant transformation of benign breast lesions is thus essential for developing effective preventive and therapeutic strategies.

Previous studies have demonstrated that angiogenesis plays a pivotal role in breast tumorigenesis and malignant progression. Consequently, various strategies have been developed to improve clinical outcomes across multiple breast cancer subtypes (Choi et al., 2025; Mohammad and Mohammad Ali, 2022). A critical process is the angiogenic switch, a hallmark event in the tumor microenvironment that marks the transition of neoplastic tissues from a dormant, avascular state to one capable of inducing neovascularization. The activation of this switch signifies a key step in tumor progression, enabling previously quiescent lesions to adopt invasive and metastatic behaviors (Pathak et al., 2024).

Tissue factor (TF), a 47-kDa transmembrane glycoprotein, is overexpressed in a variety of invasive malignancies, including breast cancer, hepatocellular carcinoma, and pancreatic cancer. Elevated TF expression has been positively correlated with increased tumor cell invasiveness and poor prognosis (Hisada and Mackman, 2019; Kamiya et al., 2026; Unruh and Horbinski, 2020). Our previous studies have demonstrated that TF-mediated activation of the angiogenic switch plays a central role in driving the progression of dormant tumors toward malignancy, as shown in an *in vivo* angiogenesis-induction model (Wu et al., 2014).

**FIGURE 1:**
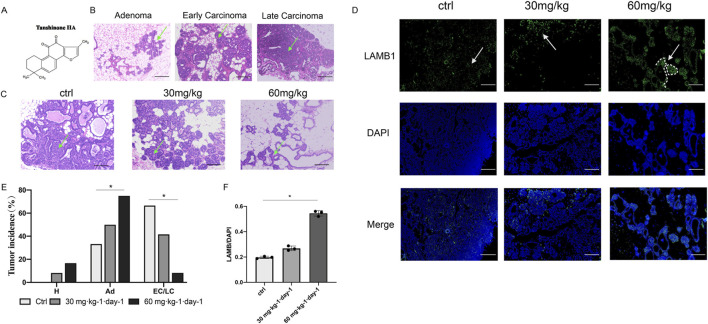
Tan IIA inhibits malignant transformation of breast tumors. **(A)** Chemical structure of Tan IIA. **(B)** Representative H&E staining images of mammary tissue from MMTV-PyMT at different stages of tumor progression. Green arrows highlight the characteristic features of malignant transformation, including the disruption of the basement membrane, nuclear enlargement, and increased cellular dispersion. Scale bar = 100 μm. **(C)** Representative H&E staining images of mammary glands from MMTV-PyMT mice treated with vehicle (Ctrl) or Tan IIA at the indicated concentrations. Green arrows indicate areas exhibiting malignant pathological changes. Scale bar = 50 μm. **(D)** Representative immunofluorescence images of Laminin subunit beta-1 (LAMB1, green)- and DAPI (nuclei, blue) staining in mammary glands. White arrows indicate the basement membrane structure. Scale bar = 50 μm. **(E)** Quantification of tumor incidence classified by the stage in each treatment group (n = 12). Abbreviations: H, hyperplasia; Ad, adenoma; EC, early carcinoma; LC, late carcinoma. Data were analyzed using Fisher’s exact test (*p < 0.05 vs. control group). **(F)** Quantitative analysis of basement membrane integrity, calculated as the ratio of the LAMB1-positive area (green) to the DAPI-positive nuclear area (blue). Data are presented as the mean ± SD. *p < 0.05 vs. control group (n = 3).

*Salvia miltiorrhiza* (danshen), a widely used botanical drug in traditional Chinese medicine (TCM), is the most frequently applied metabolite in patent formulations targeting blood stasis. Tanshinones are the major bioactive metabolites of *Salvia miltiorrhiza*, traditionally used to treat cardiovascular diseases, stroke, hyperlipidemia, arthritis, and hepatitis. Among them, Tan IIA, a fat-soluble diterpene metabolite (Figure 1A), has demonstrated significant therapeutic potential in various disease models (Ding et al., 2025; Shan et al., 2025; Zhang et al., 2025). Recent studies have revealed its anti-tumor properties, including the inhibition of proliferation, invasion, and metastasis in cancers such as ovarian, gastric, and breast cancer (Chen et al., 2025; Fang et al., 2021; Luo et al., 2025). However, the role of Tan IIA in regulating the early stages of tumor development, particularly its impact on benign-to-malignant transformation, remains largely unexplored.

In this study, we utilized a transgenic mouse model of spontaneous breast cancer mouse mammary tumor virus-polyoma virus middle T antigen (MMTV-PyMT) and a stromal gel-based angiogenesis model using dormant breast tumor cells to analyze the effects of Tan IIA on tumor progression. Specifically, we aimed to evaluate whether Tan IIA can delay the malignant transformation of benign lesions by attenuating TF-mediated activation of the angiogenic switch and elucidate its underlying molecular mechanisms.

## Results

### Tanshinone IIA delays malignant transformation from benign tumor to early carcinoma in PyMT mice

The progression of mammary epithelial lesions in MMTV-PyMT mice follows a well-defined sequence: hyperplasia (H), adenoma (Ad), early carcinoma (EC), and late carcinoma (LC). The precancerous stages, including hyperplasia and adenoma, are characterized by a normal nuclear-to-cytoplasmic (N/C) ratio and an intact basement membrane. In the adenoma stage, mammary ducts are filled with solid sheets of epithelial cells occupying the terminal ductal lobular units. In contrast, early and late carcinoma stages involve disruption of the basement membrane, nuclear enlargement, and increased cellular dispersion. At these malignant stages, the acinar structure is largely lost, and the tumor consists predominantly of solid epithelial masses (Figure 1B). Histological analysis using hematoxylin–eosin (H&E) staining and LAMB1 immunofluorescence (IF) (Figures 1C,D) revealed that in both the control group and the group treated with 30 mg/kg of Tan IIA (Figure 1B), the basement membrane structure was disrupted, and early malignant transformation was evident. However, in the 60 mg/kg Tan IIA treatment group, the mammary tissue maintained a normal N/C ratio and an intact basement membrane, consistent with the adenoma stage (Figure 1F). These findings indicate that Tan IIA, particularly at the 60 mg/kg dose, can delay the malignant progression of mammary glands from adenoma to early carcinoma in PyMT mice (Figure 1E).

### Pharmacokinetic profiling reveals that systemic exposure to tanshinone IIA and its metabolites exceeds their local accumulation in mammary tissue

**FIGURE 2:**
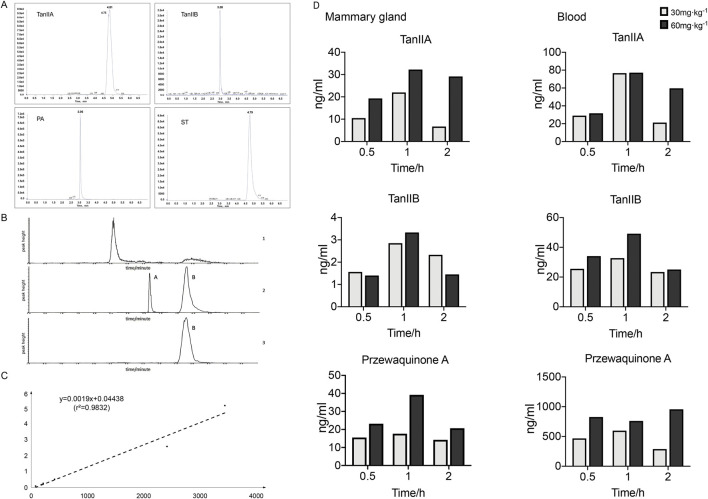
Pharmacokinetic profiling of Tan IIA and its major metabolites in plasma and mammary tissue. **(A)** Representative mass spectra of Tan IIA, its active metabolites [tanshinone IIB (Tan IIB) and przewaquinone A (PA)], and the internal control (ST, gemfibrozil). **(B)** Representative chromatograms demonstrating the specificity, separation, and detection of the analytes. The panels correspond to (1) blank plasma, (2) tanshinone IIA standard solution, and (3) plasma sample obtained 2 h after oral administration. The detected peaks are identified as **(A)** gemfibrozil (internal standard) and **(B)** tanshinone IIA. **(C)** Standard calibration curves of Tan IIA, demonstrating the linearity of the analytical method. **(D)** Concentration–time profiles of Tan IIA, Tan IIB, and PA in mouse plasma and mammary tissue following oral administration. Data represent the mean values at the indicated time-points (n = two mice per group).

Having demonstrated that Tan IIA effectively delays the malignant transformation of mammary tumors in MMTV-PyMT mice *in vivo*, we next sought to characterize its pharmacokinetic profile to better understand how it exerts these protective effects. Although Tan IIA is known for its low oral bioavailability, our study aimed to determine whether the parent metabolite and its major metabolites could be detected in both plasma and mammary tissue following oral administration. First, to ensure accurate quantitation, we established a liquid chromatography–tandem mass spectrometry (LC–MS/MS) method using gemfibrozil as an internal standard. Representative mass spectra and chromatograms confirmed the successful separation and detection of these metabolites (Figures 2A,B). Furthermore, the standard calibration curves demonstrated linearity for Tan IIA (*R* ^2^ = 0.9832; linear equation: A = 0.0019C + 0.04438), validating the reliability of our LC–MS/MS quantitation method (Figure 2C). Using this validated method, we quantified the dynamic concentrations of Tan IIA and its metabolites over time. The pharmacokinetic analysis revealed that the peak concentrations (C_max_) were reached approximately 1 h post-administration (Figure 2D). Notably, the systemic exposure levels significantly exceeded local accumulation within the mammary gland. Specifically, the highest plasma concentrations detected were 77.15 ng/mL for Tan IIA, 49.16 ng/mL for tanshinone IIB (Tan IIB), and 956.99 ng/mL for przewaquinone A (PA). In contrast, the levels in mammary tissue were markedly lower. These findings indicate that orally administered Tan IIA is rapidly metabolized and distributed, suggesting that its chemopreventive effects are likely mediated through systemic pharmacological mechanisms involving both the parent drug and its active metabolites rather than solely via direct local accumulation in breast tissue.

### Bioinformatics analysis reveals the potential mechanisms by which tanshinone IIA inhibits angiogenesis and tumor progression

**FIGURE 3:**
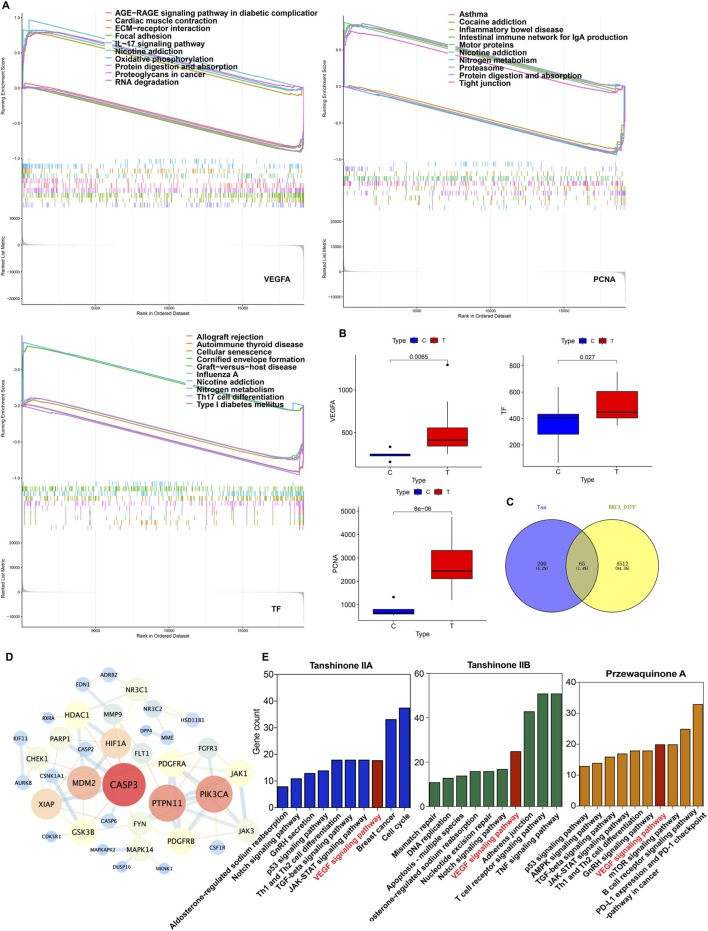
Network pharmacology and bioinformatics analysis of Tan IIA and its metabolites. **(A)** GSEA plots revealing the biological pathways associated with high expression of VEGFA, TF, and PCNA. Data were derived from the GSE21422 breast cancer dataset. **(B)** Box plots illustrating the differential expression levels of VEGFA, TF, and PCNA between breast cancer tissues and normal samples. **(C)** Venn diagram visualizing the intersection targets between the predicted potential targets of Tan IIA (including its metabolites Tan IIB and przewaquinone A) and differentially expressed genes from the breast cancer dataset GSE21422. **(D)** PPI topological network of the core intersection targets, constructed using the STRING database and visualized using Cytoscape. **(E)** KEGG pathway enrichment analysis and the corresponding signaling network highlighting the potential regulation of angiogenesis-related pathways by Tan IIA and its metabolites.

Using the GSE21422 dataset from the GEO database, we analyzed gene expression profiles from 14 breast cancer samples and normal breast tissues. Gene set enrichment analysis (GSEA) of vascular endothelial growth factor A (VEGFA), TF, and proliferating cell nuclear antigen (PCNA) revealed significant enrichment in the PPAR signaling pathway, Th17 cell differentiation, and IL-17 signaling pathway (Figure 3A). Previous studies have reported PPARγ activity in vascular tissues, particularly in endothelial cells and vascular smooth muscle cells, supporting a possible link to angiogenic regulation. Box plot analysis further confirmed that the expression levels of VEGFA, TF, and PCNA were significantly elevated in breast cancer samples compared to those of normal controls (Figure 3B), indicating their potential role in tumor proliferation and angiogenesis. To further explore the mechanism by which Tan IIA delays malignant transformation, we identified a total of 341 potential targets of Tan IIA and its active metabolites, Tan IIB and przewaquinone A, using the TCMSP and SwissTargetPrediction databases. A full list of these potential targets is provided in Supplementary Table 1. In parallel, we analyzed transcriptome data from the GSE21422 dataset to characterize the gene signatures associated with breast cancer malignant transformation (Supplementary Table 1). By intersecting the predicted drug targets with the disease-related differentially expressed genes (DEGs), we obtained a core set of overlapping genes, as visualized in the Venn diagram (Figure 3C). These overlapping targets represent the potential key mediators through which Tan IIA exerts its anti-tumor activity against breast cancer. Protein–protein interaction (PPI) networks were constructed, and key target genes were selected based on network topology parameters, such as degree, betweenness, and closeness (Figure 3D). KEGG pathway enrichment analysis revealed that Tan IIA and its metabolites may exert therapeutic effects by modulating angiogenesis-related signaling pathways, with statistical significance (*p* < 0.01) (Figure 3E).

### Tanshinone IIA inhibits angiogenesis *in vivo* in a PyMT mouse model and in a Matrigel plug assay

**FIGURE 4:**
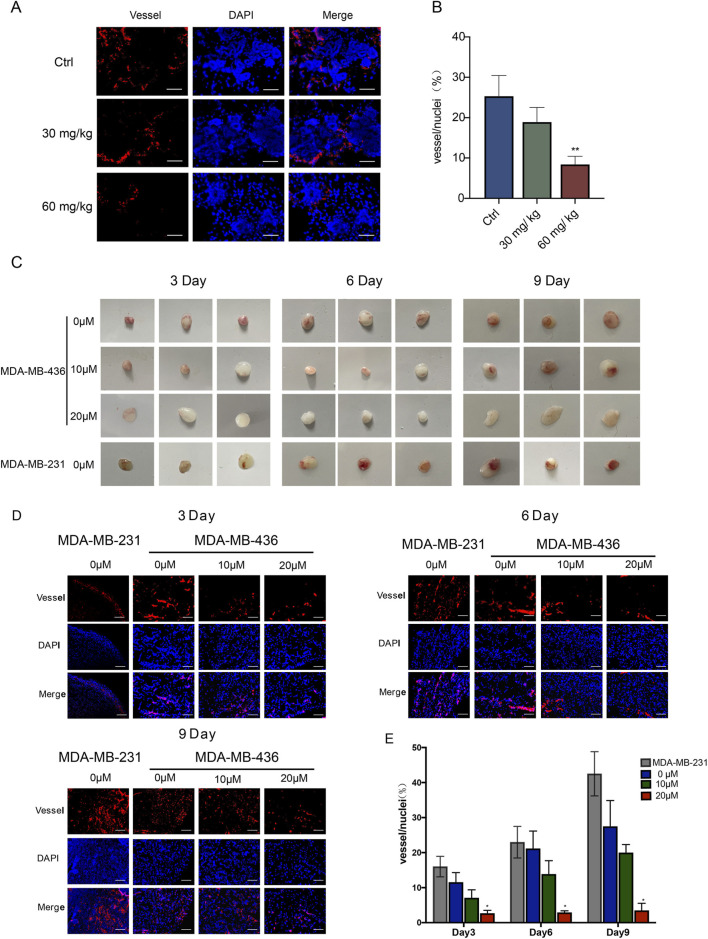
Tan IIA inhibits tumor angiogenesis *in vivo*. **(A)** Representative immunofluorescence images of mammary gland co-stained with Texas Red conjugated-dextran (red) and DAPI (blue) in the control and Tan IIA-treated groups. Scale bar = 50 μm **(B)** Quantitative analysis of blood vessels from **(A)**. Data are presented as the mean ± SD, n = 3; **p < 0.01 vs. control. **(C)** Representative gross images of Matrigel plugs harvested on days 3, 6, and 9 post-implantation (3D, 6D, and 9D, respectively). **(D)** Representative immunofluorescence images of sections of Matrigel plugs containing either control cells or tanshinone IIA-treated cells harvested at day 6 post-implantation. Scale bar = 50 μm **(E)** Quantitative analysis of blood vessels in **(D)**. Data are presented as the mean ± SD, n = 3; *p < 0.05 vs. ctrl.

Having quantified the systemic and tissue distribution of Tan IIA and its active metabolites, we next sought to evaluate whether the metabolite could inhibit angiogenesis, a process critically involved in the malignant transformation of breast tumors. To this end, we utilized two complementary *in vivo* models to assess the anti-angiogenic effects of Tan IIA. We utilized *in vivo* fluorescence labeling of blood vessels using Texas Red-conjugated dextran. In the PyMT spontaneous breast cancer model (Figures 4A,B), mice treated with 60 mg/kg Tan IIA exhibited a significant reduction in angiogenesis, as indicated by a vessel-to-nuclei ratio of 8.44 compared to 25.36 in the control group. In contrast, treatment with 30 mg/kg Tan IIA did not significantly inhibit angiogenesis (vessel/nuclei ratio = 18.95).

In a stromal thrombus-induced angiogenesis model, vessel-to-nuclei ratios were quantified on days 3, 6, and 9 post-implantation (Figures 4C–E). On day 3, the ratios were 16.01 (positive control), 11.52 (vehicle control), 7.05 (10 μM Tan IIA), and 2.64 (20 μM Tan IIA). On day 6, the values were 22.97, 21.13, 13.84, and 2.89, respectively. By day 9, the ratios increased to 42.50, 27.45, 19.95, and 3.46, respectively. These results indicate that 20 μM Tan IIA consistently and significantly suppresses angiogenesis *in vivo* across multiple time-points, further supporting its potent anti-angiogenic potential (Figure 4E).

### Tanshinone IIA suppresses the expression of pro-angiogenic factors in breast tissue

**FIGURE 5:**
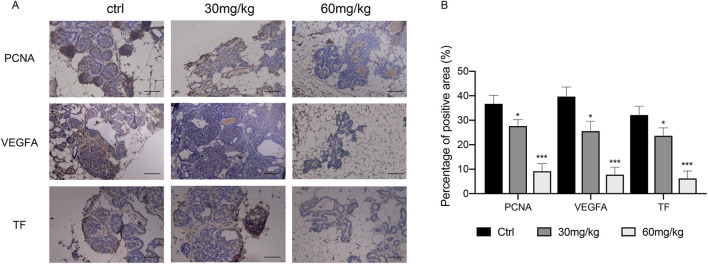
Immunohistochemical analysis of the anti-angiogenic effects of Tan IIA in PyMT mouse mammary tissue. **(A)** Representative IHC staining images of PCNA, VEGFA, and TF in mammary glands from MMTV-PyMT mice treated with or without Tan IIA. Scale bar = 50 μm. **(B)** Quantification of IHC staining intensity in **(A)**. Data are presented as the mean ± SD, n = 3. *p < 0.05 vs. control; **p < 0.001 vs. control.

To further analyze the anti-angiogenesis effects of Tan IIA, we examined the expression of PCNA, VEGFA, and TF in mammary tissue by immunohistochemistry (IHC) (Figure 5A). PCNA is a marker of tumor cell proliferation and is commonly used to assess tumor malignancy and proliferative potential. VEGFA, secreted by both endothelial cells and tumor cells, plays a central role in angiogenesis by promoting vascular formation and endothelial cell growth, and it is strongly associated with tumor progression. TF, once induced, can initiate the release of angiogenic factors through autocrine action within tumor cells or activate the angiogenic switch via paracrine signaling to surrounding stromal cells in the tumor microenvironment. Quantitative analysis of IHC staining revealed that a high dose of Tan IIA (60 mg/kg) significantly downregulated the expression of PCNA, VEGFA, and TF (Figure 5B). These findings indicate that Tan IIA at this dosage can suppress abnormal tumor cell proliferation and delay the malignant transformation into early carcinoma.

### Tanshinone IIA inhibits FGF-2-induced angiogenic factor expression *in vitro*

**FIGURE 6:**
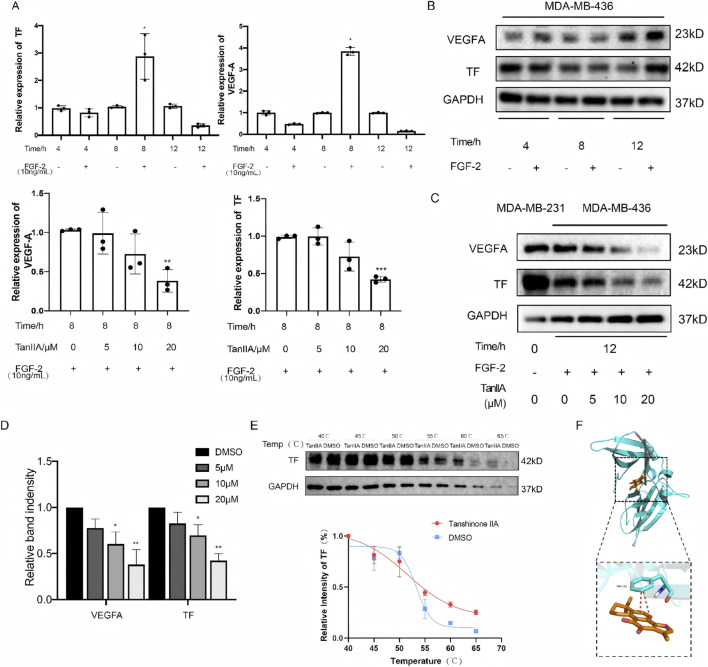
Effects of Tan IIA on angiogenesis-related gene and protein expression *in vitro*. **(A)** Real-time quantitative PCR analysis of VEGFA and TF mRNA expression in MDA-MB-436 cells treated with FGF-2 and/or Tan IIA for 4, 8, and 12 h (n = 3). **(B)** Representative Western blot analysis of VEGFA and TF protein levels in MDA-MB-436 cells following FGF-2 treatment for 4, 8, and 24 h. **(C)** Representative Western blot analysis of VEGFA and TF protein levels in MDA-MB-436 and MDA-MB-231 cells treated with or without Tan IIA for 12 h. GAPDH was used as an internal loading control. **(D)** Quantification of the densitometric ratios of VEGFA and TF protein expression from Western blots shown in **(C)** (n = 3). Data are presented as the mean ± SD. *p < 0.05 vs. control; **p < 0.001 vs. control. **(E)** CETSA–WB experiment evaluating the thermal stability of TF protein upon Tan IIA treatment (n = 3). Data are presented as the mean ± SD. **(F)** Molecular docking simulations illustrating the potential binding mode between Tan IIA and TF.

The MDA-MB-436 breast cancer cell line exhibits limited angiogenic capacity and is commonly used as a model of dormant tumor cells (Wu et al., 2014). To simulate angiogenic activation *in vitro*, avascular MDA-MB-436 cells were pre-treated with the pro-angiogenic factor fibroblast growth factor-2 (FGF-2). We determined angiogenesis-related genes *TF*, encoding tissue factor, and *VEGFA*. FGF-2 stimulation significantly upregulated *VEGFA* and *TF* at the mRNA level after 8 h and increased VEGFA and TF protein expression after 12 h, indicating a successful angiogenic switch *in vitro*. To evaluate the anti-angiogenic effect of Tan IIA, FGF-2-activated MDA-MB-436 cells were co-treated with Tan IIA. At 8 h, Tan IIA markedly inhibited the FGF-2-induced mRNA expression of *VEGFA* and *TF* (Figure 6A). Similarly, at 12 h, Tan IIA significantly suppressed their protein expression (Figures 6B–D). These results indicate that Tan IIA effectively antagonizes FGF-2-induced angiogenic activation in dormant breast cancer cells at both the transcriptional and translational levels.

To explore the potential interaction between Tan IIA and TF, we conducted a cellular thermal shift assay (CETSA) to assess the thermal stability of TF in MDA-MB-436 cells upon Tan IIA treatment. As shown in Figure 6E, Tan IIA elevated the thermal stability of TF compared with that of the vehicle control group, indicating a direct binding interaction. This interaction was further supported by *in silico* molecular docking analysis, which yielded a stable binding score of Tan IIA with TF of −6.2 kcal/mol (Figure 6F). Collectively, while further direct biophysical validation may be necessary, these preliminary results provide supporting evidence for an interaction between Tan IIA and TF, thus offering a plausible molecular context for its anti-angiogenic activity.

## Discussion

Angiogenesis is widely recognized as a hallmark of cancer and plays a central role in tumor initiation, progression, and metastasis (Acharya and Kundu, 2024; Kabir et al., 2026). As tumors grow, their increasing metabolic demands lead to hypoxic and nutrient-deprived microenvironments. Disruption of the balance between pro- and anti-angiogenic signals leads to a pathological “angiogenic switch,” driving neovascularization that supports tumor expansion (Viallard and Larrivée, 2017). Breast cancer is particularly angiogenesis-dependent, with multiple angiogenic regulators involved in the development of invasive neovessels (Mou et al., 2024; Zhou et al., 2025). Therefore, identifying effective anti-angiogenic agents is a major focus in cancer therapy.

In the present study, we verified the *in vivo* chemopreventive efficacy of Tan IIA, a natural metabolite from the botanical drug *Salvia miltiorrhiza*. Using the MMTV-PyMT mouse model, we confirmed that Tan IIA effectively delays the malignant transformation from mammary adenoma to early carcinoma. Distinct from most previous studies that explored the anti-tumor effects of Tan IIA in fully developed malignant tumors (Ge et al., 2025; Luo et al., 2025; Pathak et al., 2024), our work highlights its chemopreventive potential by attenuating TF-mediated angiogenesis during the precancerous transition stage in a mouse model of human breast cancer.

Subsequent pharmacokinetic characterization further clarified the *in vivo* distribution features of Tan IIA and its major metabolites Tan IIB and PA. Our data showed that the systemic exposure of these metabolites was markedly higher than their local accumulation in mammary tissue. Both Tan IIB and PA are well-documented bioactive metabolites of *Salvia miltiorrhiza*. Tan IIB exerts antioxidant, anti-inflammatory, and anti-tumor effects, while PA can inhibit tumor cell proliferation, induce autophagy, and regulate cell-cycle progression (Su et al., 2026). These findings suggest that the anti-angiogenic and anti-malignant transformation effects of Tan IIA are mainly mediated through systemic actions and that circulating metabolites are likely to participate in the overall pharmacological response. This part of the data clarifies the material basis for the *in vivo* efficacy of Tan IIA and its metabolites and rules out the possibility that the therapeutic effect solely relies on local accumulation within mammary tissue. We hypothesize that circulating metabolites may indirectly regulate mammary angiogenesis by targeting tumor-associated endothelial cells or remodeling the systemic inflammatory microenvironment (Zhang et al., 2022). Nevertheless, the direct causal relationship between systemic metabolite exposure and local anti-angiogenic effects has not been fully validated in this study, which is acknowledged as a limitation of this work.

To further dissect the underlying molecular mechanism, we conducted network pharmacology and bioinformatics analysis to screen the core signaling pathways associated with Tan IIA intervention. GSEA and KEGG pathway enrichment revealed that the targets of Tan IIA and its metabolites were significantly enriched in angiogenesis-related cascades, among which the VEGF signaling pathway was identified as the core regulatory axis. In addition, the PPAR and IL-17 signaling pathways were also closely correlated with the hyperactive angiogenic state in breast cancer, consistent with the reported regulatory function of PPARγ in vascular tissues (Kanehisa et al., 2025). These bioinformatic results provide directional hypotheses for subsequent mechanistic verification and indicate that Tan IIA acts via a multi-target multi-pathway synergistic network to inhibit angiogenesis. It should be noted that network pharmacology only generates predictive hypotheses rather than definitive conclusions, and all inferences require rigorous experimental validation.

Guided by the pathway screening results, we further focused on key functional proteins within the VEGF-related angiogenic pathway and carried out a series of *in vivo* and *in vitro* mechanistic studies. While our study demonstrates that Tan IIA acts systemically rather than via direct mammary accumulation, we acknowledge concerns regarding potential off-target effects. The 60 mg/kg dose was selected based on established preclinical data showing no overt toxicity in major organs (Ren et al., 2010; Xuan et al., 2023). Tan IIA likely targets tumor-specific pathways (e.g., angiogenesis) that are less active in healthy tissues, thus reducing off-target risks. Future studies will explore targeted delivery systems to enhance tumor accumulation and minimize exposure to healthy tissue. Specifically, the high plasma concentration of przewaquinone A requires further analysis to determine whether it is a primary active metabolite contributing to the observed efficacy.

TF and VEGFA are two critical molecules involved in this cascade. *In vivo* experiments confirmed that high-dose Tan IIA significantly downregulated the expression of TF and VEGFA in mammary tissue, along with reduced expression of the proliferation marker PCNA. Two independent *in vivo* angiogenesis models also verified the potent anti-angiogenic capacity of Tan IIA. *In vitro* assays using FGF-2-activated dormant breast cancer cells further proved that Tan IIA could inhibit the upregulation of TF and VEGFA at both the transcriptional and translational levels. Since TF acts upstream in the angiogenic cascade to trigger the angiogenic switch and elevate VEGFA expression via autocrine and paracrine signaling, we first examined the direct interaction between Tan IIA and TF using CETSA and molecular docking. These approaches provide biophysical evidence of a potential direct interaction between Tan IIA and TF, thereby indicating a thermodynamically favorable binding mode that supports the formation of a stable complex. While these *in vitro* findings support the notion of physical binding, the definitive functional relevance and causal effects of this interaction *in vivo* await further validation through targeted TF modulation assays.

As a natural metabolite of the botanical drug *Salvia miltiorrhiza*, Tan IIA likely exerts chemopreventive activity via synergistic regulation of multiple molecular targets rather than modulation of a single signaling pathway. Our previous gene manipulation studies have established the pivotal role of TF in driving breast cancer angiogenesis (Wu et al., 2014). Consistent with this finding, our *in vivo* data show that Tan IIA effectively suppresses TF-mediated angiogenic progression. Nevertheless, apart from TF and VEGFA, other predicted targets identified through our network analysis may also participate in the overall anti-tumor process, and their synergistic contributions cannot be excluded. Furthermore, the respective roles of its circulating metabolites, Tan IIB and PA, in suppressing angiogenesis and tumor progression have not been systematically verified through independent *in vitro* and *in vivo* studies.

Despite the observed inhibitory effect of Tan IIA on the malignant transformation of benign mammary tumors via regulating vascular permeability, the present study has several limitations: first, the duration of the *in vivo* experiments is relatively short, which restricts the dynamic observation of vascular remodeling and tumor progression. The *in vivo* experiment duration was set to 5 weeks. Although this duration can reflect short-term changes in tumor and vascular-related phenotypes, it may not fully capture the long-term mechanisms of tumor progression and the dynamic process of vascular remodeling, which limits the interpretation of the long-term effectiveness of the study results to a certain extent. We plan to incorporate a dorsal skinfold chamber model to enable real-time visualization of vasculature, along with IVIS imaging to scan tumors and demonstrate fluorescence accumulation over time in our subsequent studies, which would significantly enhance the depth and reliability of our findings; second, the sample sizes for the pharmacokinetic profiling are relatively small (n = 2 per group). Given the modeling cycle and limited availability of specific-stage MMTV-PyMT mice, this part was designed as an exploratory pilot evaluation. While the intra-group consistency provides preliminary evidence for systemic exposure, definitive pharmacokinetic parameters await further validation in larger cohorts using standard animal models (e.g., C57BL/6 mice). Third, the standard calibration curve for Tan IIA was established using a solvent matrix rather than separate biological matrices (serum and mammary tissue), which we will optimize in subsequent targeted pharmacokinetic studies.

## Conclusion

In conclusion, our integrated pharmacological and mechanistic study suggests that Tan IIA is a potent inhibitor of the TF-driven angiogenic switch. By suppressing this critical initiation step, Tan IIA effectively delays the malignant transformation of benign breast lesions into invasive carcinoma in a clinically relevant spontaneous model. The metabolite’s unique pharmacokinetic profile, characterized by higher systemic than local exposure, opens new avenues for research into its mode of action, potentially involving systemic modulation or bioactive metabolites. Our findings underscore the translational potential of Tan IIA as a novel natural product-based strategy for the prevention of breast cancer progression, thus offering a promising complementary approach to current therapeutic paradigms.

## Materials and methods

### Reagents

Fetal bovine serum (FBS; Cat# SA311.02) was purchased from CellMax (Beijing, China). L-15 medium (Cat# CM-0383) was purchased from Procell (Wuhan, China). Dulbecco’s modified Eagle medium (DMEM; Cat# KGM12800-500) was purchased from KeyGEN BioTECH (Nanjing, China). Dimethyl sulfoxide (DMSO; Cat# C12007439) was purchased from Macklin, and an additional DMSO stock (Cat# D5879-500 ML) was purchased from Sigma-Aldrich (Darmstadt, Germany). Goat anti-rabbit IgG (H + L) HRP (Cat# BS13278) and goat anti-mouse IgG (H + L) horseradish peroxidase (HRP) (Cat# BS12478) were purchased from Bioworlde (Minnesota, United States). Lysine-fixable, Texas Red-conjugated dextran (70,000 MW) was purchased from Thermo Fisher Scientific (D1864, Waltham, United States). Matrigel Matrix (Cat# 356231) was purchased from Corning (New York, United States). Phenylmethylsulfonyl fluoride (PMSF; Cat# ST506) was purchased from Beyotime Biotechnology (Shanghai, China). Reagents for RNA and qPCR analysis, including RNA Isolater Total RNA Extraction Reagent (Cat# R401-01) and HiScript II Q RT SuperMix for qPCR (Cat# R223-01), and ChamQ SYBR qPCR Master Mix (Cat# Q331-02) were purchased from Vazyme (Nanjing, China). The immunohistochemistry kit (Cat# PV-9000) was purchased from Zhongshan Golden Bridge Biotechnology (Beijing, China). Primer antibodies were as follows: anti-vascular endothelial growth factor A (VEGFA; Cat# ab1316) and anti-tissue factor (TF; Cat# ab211016) were purchased from Abcam (Cambridge, United States). Anti-proliferating cell nuclear antigen (PCNA; Cat# AF0239) was purchased from Affinity Biosciences (Melbourne, Australia); anti-Laminin beta 1 Rabbit polyclonal Ab (Cat# A9827) was purchased from Abclonal (Wuhan, China). Fibroblast growth factor-2 (FGF-2; Cat#AF-100-18B) was purchased from PeproTech (New Jersey, United States). Tanshinone IIA (Cat# B20257-200 mg, purity ≥ 98% by high-performance liquid chromatography (HPLC)) was purchased from Yuanye Biotechnology (Shanghai, China).

### Cell culture and tanshinone IIA treatment

The breast cancer lines MDA-MB-436 and MDA-MB-231 were obtained from Cell Bank of the Chinese Academy of Sciences. Cells were cultured according to the supplier’s recommendations. MDA-MB-436 cells were cultured in Leibovitz’s L-15 medium supplemented with 10 μg/mL insulin, 16 μg/mL glutathione, 15% FBS, and 1% penicillin–streptomycin (P/S) under a humidified atmosphere of 100% air. MDA-MB-231 cells were cultured in DMEM supplemented with 10% FBS under a 5% CO_2_ and 95% air-humidified atmosphere. Tan IIA was dissolved in DMSO to form a stock concentration of 20 mM and stored at −20 °C.

### Animal studies

Polyoma virus middle T antigen (PyMT) mice were obtained from Nanjing GemPharmatech (Nanjing, China) and backcrossed to mice with a C57BL/6 background for 10 generations. Female BALB/c nude mice (7–8 weeks old) were supplied by Shanghai SLAC Laboratory Animal Co., Ltd. (Shanghai, China). Mice were housed under specific pathogen-free and temperature-controlled conditions. All animal experiments were conducted in accordance with the institutional guidelines of Nanjing University of Chinese Medicine and approved by the Animal Ethics Committee of Nanjing University of Chinese Medicine (approval number: 202105A022). The study is reported in accordance with the ARRIVE guidelines. PyMT mice were divided randomly into three groups, namely, the control group, Tan IIA 30 mg/kg group, and Tan IIA 60 mg/kg group (n = 6 per group). The control group was administered olive oil alone, and the Tan IIA groups were given daily oral gavage of Tan IIA in olive oil at doses of 30 and 60 mg/kg for 5 consecutive weeks. Nude mice were divided randomly into four groups, namely, the control group, positive group, Tan IIA 10 µM group, and Tan IIA 20 µM group (n = 3 per group).

### Immunofluorescence staining

Paraffin-embedded tissue sections were prepared from samples fixed overnight at 4 °C in 4% paraformaldehyde (PFA). After fixation for 24 h, standard dehydration, paraffin embedding, sectioning, and rehydration were carried out. Antigen retrieval was performed using 10 mM sodium citrate buffer at 95 °C for 15 min. Sections were blocked with 5% BSA for 1 h and incubated overnight at 4 °C with laminin β1 antibodies (1:1,000 dilution in 5% BSA in PBS) (A9827, Abclonal). After three washes with PBS, sections were incubated with the FITC-labeled secondary antibody (1:1,000) (Ab6717, Abcam) in PBS for 1 h in the dark at room temperature. Nuclei were counterstained with 4′,6-diamidino-2-phenylindole (DAPI), and images were captured using a ZEISS microscope (Oberkochen, Germany) or Leica SP8 confocal microscopy (Heidelberg, Germany).

### Immunohistochemistry staining

After fixation, paraffin embedding, and rehydration, tissue sections underwent heat-mediated antigen retrieval. Endogenous peroxidase activity was blocked with 3% hydrogen peroxide. Sections were incubated overnight with antibodies against VEGFA (1:100) (ab1316, Abcam), TF (1:1000) (ab318995, Abcam), and PCNA (1:1000) (AF0239, Affinity Biosciences), followed by HRP-conjugated secondary antibodies. Detection was performed using 3,3′-diaminobenzidine (DAB), counterstained with hematoxylin, sealed with neutral gum, and observed with a Pathology Workstation (Mantra, PerkinElmer).

### Liquid chromatography/tandem mass spectrometry analysis

Breast tissue samples (200 µg) were mixed with 400 µL of 50% internal standard in methanol. Homogenates were centrifuged at 12,000 rpm for 10 min, and supernatants were collected. Gemfibrozil (20 µL, 44 μg/mL) was added, and samples were vortexed and centrifuged again. Supernatants were evaporated to dryness using a refrigerated concentrator and reconstituted in 200 µL of 50% acetonitrile before LC–MS/MS analysis (Q-TRAP 4500). Plasma samples followed a similar protocol. Standard concentrations for Tan IIA ranged from 1.1 to 296 ng/mL.

### *In vivo* fluorescence labeling of blood vessels

Texas Red-labeled dextran (70,000 MW, 21 μg/g body weight) (D1864, Invitrogen) was injected into the tail vein of mice 5 min–7 min prior to tissue harvesting. Tissues were then fixed, embedded in paraffin, and counterstained with DAPI. Vessels were visualized using fluorescence microscopy. The methods followed previously published protocols from our group (Wu et al., 2014).

### Matrigel plug assay

Avascular human breast cancer cell line MDA-MB-436 cells were treated with Tan IIA (10 µM or 20 µM) for 24 h. Treated cells (5 × 10^5^) were resuspended in 30 µL culture medium, mixed with 70 µL Matrigel (Cat # 356231, Corning), and injected into the abdominal mammary glands of female nude mice. The breast cancer cell line MDA-MB-231 was used as the positive control due to its highly angiogenic potential.

### Real-time qPCR

Total RNA was isolated using Total RNA Extraction Reagent (R401-01-AA, Vazyme, Nanjing, China). Then, cDNA was synthesized from 500 ng total RNA by reverse transcription using the Veriti™ 96-Well Thermal Cycler (Applied Biosystems). Quantitative PCR (Q-PCR) was carried out with the Life Technologies 7500 Real-Time PCR System (Applied Biosystems) using ChamQ Universal SYBR qPCR Master Mix (Q711-02, Vazyme, Nanjing, China). Data were analyzed using the 2^−ΔΔCt^ method. The primer sequences were listed as follows:

*GAPDH*-F: (5′- GGA​GCG​AGA​TCC​CTC​CAA​AAT - 3′).
*GAPDH*-R: (5′- GGC​TGT​TGT​CAT​ACT​TCT​CAT​GG - 3′).
*VEGFA*-F: (5′- AGG​GCA​GAA​TCA​TCA​CGA​AGT - 3′).
*VEGFA*-R: (5′- AGG​GTC​TCG​ATT​GGA​TGG​CA - 3′).
*TF*-F: (5′- GGC​GCT​TCA​GGC​ACT​ACA​A - 3′).
*TF*-R: (5′- TTG​ATT​GAC​GGG​TTT​GGG​TTC - 3′).

### Western blot

Cells were lysed using RIPA buffer containing a protease inhibitor cocktail, phenylmethylsulfonyl fluoride (PMSF). Lysates were centrifuged at 12,000 rpm for 15 min at 4 °C. A total of 20 μg protein samples for each group were separated by PAGE and transferred to PVDF membranes. Primary antibodies: anti-VEGFA antibody was diluted in the ratio 1:100, and anti-TF antibody was diluted in the ratio 1:1,000. The secondary antibodies were goat anti-rabbit IgG (H + L) HRP (1:10,000 dilution) and goat anti-mouse IgG (H + L) HRP (1:10,000 dilution). Finally, the bands were detected using a gel imaging system (Bio-Rad, California, United States).

### Cellular thermal shift assay

The interaction between Tan IIA and TF was evaluated using the CETSA. The soluble protein lysate of MDA-MB-436 cells was aliquoted into tubes and treated with Tan IIA (100 μmol/L) or DMSO for 2 h at RT prior to CETSA. The solutions were heated at the indicated temperatures (40 °C–65 °C) for 3 min, followed by cooling at 4 °C for 3 min in a thermocycler (Applied Biosystems, United States). After centrifugation for 20 min (15,000 ×g, 4 °C), the soluble supernatant was subjected to Western blotting.

### Molecular docking

The chemical structure of Tan IIA was retrieved from the PubChem database (https://pubchem.ncbi.nlm.nih.gov/, access date: February 18, 2025) with metabolite CID: 164676. The three-dimensional structure of TF (PDB ID: 1BOY) was obtained from the Protein Data Bank (PDB) database (https://www.rcsb.org/, access date: February 18, 2025) and supplemented by AlphaFold predicted structures. The protein preparation was conducted using AutoDock Tools, which included the sequential execution of preprocessing steps and charge assignment. The processed structures were exported in the PDBQT format. For AutoDock Vina docking, the docking center of the protein obtained from the PDB database was defined at the binding site of the co-crystallized small-molecule inhibitor within the TF protein. The binding affinity was quantified based on the scoring of the docking conformations and intermolecular interactions between Tan IIA and TF protein. Finally, the 3D diagram of the protein-small molecule docking was generated using PyMOL.

### Network pharmacology and target prediction

The potential targets of Tan IIA, Tan IIB, and przewaquinone A were predicted using the TCMSP database (https://old.tcmsp-e.com/tcmsp.php, access date: January 6, 2025), filtered by oral bioavailability (OB) ≥ 30% and drug-likeness (DL) ≥ 0.18, along with the SwissTargetPrediction database (www.swisstargetprediction.ch, access date: January 6, 2025). In parallel, the gene expression profiles were retrieved from the Gene Expression Omnibus (GEO) database (https://www.ncbi.nlm.nih.gov/geo/, access date: February 20, 2025) (GSE21422). DEGs were identified by comparing breast cancer tissues with normal tissue samples (Supplementary Table S1). Subsequently, the intersection between the predicted drug targets and these disease-related DEGs was identified and visualized using a Venn diagram to determine the potential therapeutic targets of Tan IIA against breast cancer.

### Protein–protein interaction network construction and pathway enrichment

The PPI network was analyzed using the STRING database (https://string-db.org/, access date: January 6, 2025) by importing the identified targets. Only interactions with a confidence score ≥0.7 were selected to establish the PPI network. Identified target proteins that were independent of the network were removed. The network thus obtained was visualized and analyzed using Cytoscape (v3.9.1).

### Gene set enrichment analysis

The clinically relevant gene expression profile dataset GSE21422 was extracted from the GEO database. The dataset included data from 14 primary and metastatic tumor specimens and five healthy samples. GSEA was performed using the clusterProfiler package to evaluate the enrichment of pathways associated with *VEGFA*, *TF*, and *PCNA*. Statistical significance for all the bioinformatics analyses was defined as *p* < 0.01.

## Data Availability

The datasets presented in this study are available in public repositories. The GEO dataset analyzed in this work can be accessed under accession number [GSE21422], and all other raw data are available from the corresponding author upon reasonable request.

## Glossary

Ad: Adenoma
CETSA: Cellular thermal shift assay
**C**_**max**_: Peak concentration
DAB: 3,3′-Diaminobenzidine
DAPI: 4′,6-Diamidino-2-phenylindole
DEGs: Differentially expressed genes
DL: Drug-likeness
DMEM: Dulbecco’s modified Eagle medium
DMSO: Dimethyl sulfoxide
EC: Early carcinoma
FBS: Fetal bovine serum
FGF-2: Fibroblast growth factor-2
GEO: Gene Expression Omnibus
GSEA: Gene set enrichment analysis
H: Hyperplasia
H&E: Hematoxylin–eosin
HRP: Horseradish peroxidase
IF: Immunofluorescence
IHC: Immunohistochemistry
LAMB1: Laminin subunit beta-1
LC: Late carcinoma
LC–MS/MS: Liquid chromatography–tandem mass spectrometry
N/C ratio: Nuclear-to-cytoplasmic ratio
OB: Oral bioavailability
PA: Przewaquinone A
PCNA: Proliferating cell nuclear antigen
PDB: Protein Data Bank
PFA: Paraformaldehyde
PMSF: Phenylmethylsulfonyl fluoride
PPI: Protein–protein interaction
PyMT: Polyoma virus middle T antigen
qPCR: Quantitative polymerase chain reaction
RT: Room temperature
Tan IIA: Tanshinone IIA
Tan IIB: Tanshinone IIB
TCM: Traditional Chinese medicine
TF: Tissue factor
VEGFA: Vascular endothelial growth factor A
WB: Western blot
